# In Vivo Evaluation of the Potential of Thyme and Lemon Hydrolates as Processing Aids to Reduce Norovirus Concentration during Oyster Depuration

**DOI:** 10.3390/foods12213976

**Published:** 2023-10-30

**Authors:** Roberta Battistini, Chiara Masotti, Daniela Manila Bianchi, Lucia Decastelli, Aitor Garcia-Vozmediano, Cristiana Maurella, Marie-Laure Fauconnier, Antonello Paparella, Laura Serracca

**Affiliations:** 1 Istituto Zooprofilattico Sperimentale del Piemonte Liguria e Valle d’Aosta, 10154 Turin, Italy; roberta.battistini@izsto.it (R.B.); manila.bianchi@izsto.it (D.M.B.); lucia.decastelli@izsto.it (L.D.); aitor.garciavozmediano@izsto.it (A.G.-V.); cristiana.maurella@izsto.it (C.M.); laura.serracca@izsto.it (L.S.); 2 Laboratory of Chemistry of Natural Molecules, Gembloux Agro-Bio Tech, University of Liège, Passage des Déportés 2, 5030 Gembloux, Belgium; marie-laure.fauconnier@uliege.be; 3 Department of Bioscience and Technology for Food, Agriculture and Environment, University of Teramo, Via R. Balzarini 1, 64100 Teramo, Italy; apaparella@unite.it

**Keywords:** *Crassostrea gigas*, post-harvest processing, depuration, natural compounds, norovirus

## Abstract

In this study, we evaluated the use of hydrolates, co-products of essential oil distillation, as processing aids to improve the depuration process of Pacific oysters (*Crassostrea gigas*) as a post-harvest method aimed at reducing the norovirus (NoV) viral load. Live oysters were kept in water to which hydrolates of *Thymus serpyllum* and *Citrus limon* at 1% were added for 24 h. The concentration of NoV was quantified using the ISO 15216-1 quantitative real-time RT-PCR method in the oyster digestive tissue both before and after the treatment. The results showed a significant reduction of 0.2 log in the NoV GII concentration after 24 h of treatment with 1% *C. limon* hydrolate. Conversely, treatment with *T. serpyllum* did not appear to reduce the concentration of NoV compared to the control. Additionally, a sensory analysis was conducted through a blind survey comparing untreated and treated oysters. No changes in the sensory and physical characteristics of the oysters were observed, except for a decrease in the marine flavour intensity, which was positively perceived by consumers. These results indicate that the addition of hydrolates of *C. limon* at 1% during depuration might represent a promising processing aid for enhancing both the safety and acceptability of live oysters.

## 1. Introduction

Oysters are an economically important seafood that is consumed worldwide, and the species *Crassostrea gigas* is the most widely produced [1]. Oyster aquaculture production has rapidly expanded over recent decades, and this could increase the public health risk associated with the consumption of these products, since oysters concentrate pathogens during their filtration activity and are usually consumed raw. The primary contamination source of bivalve mollusks often occurs at the stage of primary production, during their filter feeding in water contaminated with pathogenic microorganisms [2,3]. norovirus (NoV) is one of the major pathogens that can contaminate oysters, and has often been linked to seafood-related outbreaks [4,5,6,7,8,9,10]. NoV can be genetically classified into ten different genogroups (GI-GX), which can be further subdivided into different genetic groups or genotypes [11]. Only genogroups I, II, and IV infect humans, and NoV commonly isolated in cases of acute gastroenteritis belongs to GI and GII; moreover, GII genotype 4 accounts for the majority of cases of gastroenteritis in adults, and is often present worldwide [6]. In 2021, NoV ranked as the third most frequently reported causative agent in foodborne outbreaks in the EU, with 147 human outbreaks associated mainly with the consumption of crustaceans, mollusks, and derivatives [12]. Depuration is usually the main treatment used for shellfish growing in class B farming areas according to EU legislation. However, this post-harvest processing technique is considered inconsistent for reducing NoV in live oysters, due to the interaction of these pathogens with particular ligands that are present in the oyster tissues [2,13]. Traditional heat treatment is highly efficient in inactivating noroviruses, but this process alters the sensory properties of foods and is unsuitable for oysters that are typically eaten raw [14,15]. Several alternative post-harvest methods have been proposed [16], including chemical sanitizers [17,18,19], high pressure processing (HPP) [20], cold plasma [21], and gamma irradiation [22,23]. These alternatives offer advantages but also have limitations. In fact, chemical sanitizers are potentially toxic and may induce adverse health effects [17,18,19], whereas HPP efficacy against foodborne viruses depends on various factors including processing parameters, virus type, and food matrix [20]. Regarding cold plasma, recent studies on human norovirus in seafood have yielded contrasting results, as the antiviral efficacy of plasma appears to be related to the treatment parameters and the plasma system used [21,24]. Finally, gamma irradiation requires complex equipment, and is very expensive and hazardous for exposed workers [22]. Thus, there is the need to identify new post-harvest treatments that are safe, efficient, practical, and convenient for NoV inactivation in shellfish. Based on these characteristics, the use of natural antimicrobials during oyster depuration could be a promising solution.

Plant extracts can provide unlimited opportunities for microbial control, thanks to their large chemical diversity [25,26,27]. In recent years, several studies have explored the antimicrobial properties of essential oils (EOs) and/or their active components against microorganisms present in foods [28,29,30]. However, practical applications of EOs face significant challenges, due to their strong hydrophobic nature, which hinders their dispersion in liquids, and their potent odor.

By contrasts, hydrolates, or aromatic waters, are aqueous solutions obtained as by-products during the production of EOs through the steam distillation or hydrodistillation of fresh medicinal plants. Recently, these compounds have garnered attention in the food industry due to their antibacterial, antifungal, and antioxidant properties similar to the EOs they are derived from [31,32]. Additionally, thyme and lemon hydrolates have exhibited promising virucidal effects in vitro against murine norovirus (MNV), a human norovirus surrogate [33]. In fact, these hydrolates contain as their main components substances with demonstrated antiviral activity such as carvacrol and limonene [34,35,36,37]. Hydrolates are cost-effective, easily obtainable, and less toxic than EOs. Being aqueous solutions, they can be readily dispersed in water without the strong odor of EOs. To date, research on hydrolates as an antiviral approach in shellfish and against NoV has not been reported. From a regulatory perspective, hydrolates can be used as “processing aids” under Regulation (EC) 1169/2011 on food labelling, provided they serve a specific technological purpose in processing and their residues have neither health risks nor technological effects on the final products [38]. The aim of this study was to investigate the potential use of *Thymus serpyllum* and *Citrus limon* hydrolates as processing aids to improve the depuration efficacy of Pacific oysters for reducing NoV concentration, also evaluating the potential sensory impact on the shellfish.

## 2. Materials and Methods

### 2.1. Experimental Design

*Crassostrea gigas* oysters naturally contaminated with NoV in their growing areas (Class B) were used to evaluate the virucidal activity of *Thymus serpyllum* and *Citrus limon* hydrolates. In each experiment, a batch of 40 oysters was harvested from the same growing area and acclimatized for 24 h in a tank containing 30 liters of seawater, maintained at room temperature (19–20 °C) and aerated using an aquarium pump. Subsequently, the oysters were transferred into an identical tank with clean seawater supplemented with hydrosols of *T. serpyllum* or *C. limon* at a concentration of 1% (*v*/*v*). The oysters were maintained in this seawater, to which each hydrolate was added, for 24 h, during which time they carried out their normal filtration activity. Both at the beginning of the experiment (t = 0) and after 24 h of treatment, we sampled 10 oysters for NoV analysis. As a negative control, 10 oysters were maintained in seawater without the presence of hydrolates for 24 h. Experiments were performed in triplicate. The concentration of hydrolates used in this study was determined based on previous experiments [33]. The same experiments were repeated on oysters that resulted negative to NoV for sensory evaluation by a consumer panel.

### 2.2. Hydrolates

*Thymus serpyllum* (thyme) and *Citrus limon* (lemon) hydrolates supplied by I Segreti delle Erbe (Netro, Bl, Italy) were used in this study. These hydrolates were analyzed through SPME-GC-MS using a 50/30 μm DVB/CAR/PDMS (Supelco, Bellefonte, PA, USA) fiber that was preconditioned according to the instructions of the manufacturer. Extraction was performed by immersing the fiber directly into the hydrolates that had been preheated to 30 °C for 1 min. The chromatographic conditions were set as follows: injection mode: splitless at 280 °C; HP-5MS capillary column (Agilent, Santa Clara, CA, USA) (30 m × 0.25 mm, df = 0.25 μm); temperature program: from 40 °C (2 min) to 300 °C (5 min) at a rate of 6 °C/min. Helium served as the carrier gas at a flow rate of 1.2 mL/min. Mass spectra were recorded in electron ionization mode at 70 eV (scanned mass range: 35–400 *m*/*z*). The source and quadrupole temperatures were set at 230 °C and 150 °C, respectively. The compound identification was based on chromatographic retention indices (RI) and the comparison of the recorded spectra with a computed data library (Pal 600K^®^). The experimental retention index (RI) of the compounds was calculated by injecting a mixture of n-alkanes C8–C20 (Sigma Aldrich, Darmstadt, Germany). The results were expressed as a percentage of the total chromatographic area.

### 2.3. Detection and Quantification of Norovirus

Oyster samples were analyzed for NoV according to the ISO 15216-1:2017/A1:2021 method [39], as described in the following paragraphs.

#### 2.3.1. Virus Extraction

The hepatopancreas of a minimum of 10 mollusks was excised for each oyster sample, and finely chopped. To two grams of this homogenate were added 10 μL of mengovirus (MV) (used as extraction process control) and 2 mL of proteinase K solution (0.1 mg/mL). After vigorously mixing the tubes to uniformly distribute the contents, the samples were subjected to a first incubation at 37 °C with constant stirring for 60 min, followed by a second incubation in a water bath at 60 °C for 15 min. Subsequently, we centrifuged the samples at 3000× *g* for 5 min, transferred the supernatants to new tubes, and recorded the final volume. The supernatants were then stored at −20 °C until RNA extraction was performed.

#### 2.3.2. Nucleic Acid Extraction

Viral RNA was extracted from 500 μL of the shellfish supernatants using the EGENE-UP^®^ platform and the NucliSens magnetic extraction reagents (bioMérieux, Marcy l’Etoile, France) following the manufacturer’s instructions. RNA was eluted into 100 μL of elution buffer and stored at −80 °C until testing.

##### Norovirus Quantification

For NoV GGI and GGII, real-time reverse transcription-PCR amplification was performed using a C1000 Thermal Cycler (Biorad Laboratories, Hercules, CA, USA) and the RNA Ultrasense one-step kit qRT- PCR (Invitrogen, Life Technologies, Waltham, MA, USA). For both NoV genogroups, 5 μL of sample was added to 20 μL of mixes containing 1 X of Ultrasense reaction mix, 500 nM of the forward primer and 900 nM of the reverse primer, 250 nM of the probe, 1.25 μL of RNA Ultrasense enzyme mix, and ultrapure water by volume. The primers and probes used were in accordance with ISO 15216-1:2017/A1:2021 [39]. Reverse transcription was performed at 55 °C for 60 min, followed by preheating to 95 °C for 5 min, and 45 cycles of PCR (denaturation at 95 °C for 15 s, annealing at 60 °C for 60 s, and extending at 65 °C for 60 s). Negative and positive controls were included in each plate to monitor the correctness of the assay. Plasmid RNA of the various targets was used as a positive control to verify the amplification efficiency, and the RNA of MV after virus extraction was used as a control of the extraction efficiency. Standard curves for NoV quantification were created using the tenfold serial dilution of a dsDNA standard for NoV GI and NoV GII. Consequently, the number of NoV RNA copies in each sample was determined by comparing the Ct value to the standard curves. The final concentration was then calculated based on the volume of the analyzed extract, and expressed as genomic copies per gram (g.c./g). All samples were evaluated for extraction efficiency by comparing the Ct value of MV obtained in spiked samples with a standard curve derived from the extraction of 10 μL of MV. Samples with extraction efficiency greater than 1% were deemed acceptable. The presence of RT-qPCR inhibitors was evaluated by adding 1 μL of external control (EC) RNA for each genogroup. The Ct value obtained in samples spiked with the EC RNA was then compared to that of water spiked with the EC RNA. Inhibition values below 75% were considered valid. Curves with amplification efficiencies of ~90% to 110% were used for calculations. The limit of quantification (LOQ) for NoV GI and NoV GII assays was 140 g.c./g and 130 g.c./g, respectively. The limit of detection of 50% (LOD_50_) was 7.8 g.c./g for NoV GI and 12.4 g.c./g for NoV GII.

### 2.4. Sensory Evaluation

We conducted a sensory analysis on oysters, both treated with hydrolates and untreated, using a panel of 36 volunteers. The participants were selected based on their status as oyster consumers without allergies to oysters, lemon, or thyme. Most of the volunteer tasters were male (20 male and 16 female) and were generally above 50 years of age (approx. between 25 and 65). The tasters participated voluntarily without compensation and were unaware of the study’s objectives or experimental design. The sensory evaluation involved a total of 120 oysters, divided into three groups of 40 oysters each, according to the treatment status (referred to as Group A, Group B, and Group C). Following hydrolate treatment, all three groups of oysters underwent the classic purification treatment in the relaying plants for 24 h before sensory evaluation. The oysters were kept refrigerated until preparation for the consumer test. The oysters were manually shucked and the tissue was placed back on the lower shell. Tests were carried out at room temperature. The tasters were seated at random and were instructed not to communicate to each other during or between the evaluations. Each taster was presented with three blinded oysters: oyster A was an untreated oyster, oyster B was treated with *C. limon* hydrosol, and oyster C with *T. serpyllum* hydrosol. A new sample, consisting of a half-shell oyster on a plastic plate, was presented to each taster every 10 min, during which time they could drink water. The tasters were asked to fill in the respective panel test cards and to express a preference based on appearance, smell, and taste (orthonasal and retronasal perception) among the three different samples. In detail, the attributes for the sensory profile test included overall color, opacity, marine odor, citrus odor, garlic odor, rosemary odor, thyme odor, saltiness, sweetness, bitterness, sourness, marine taste, algal taste, raspberry taste, metallic taste, thyme taste, spinach taste, lemon taste, chewiness, overall acceptability, and, finally, favorite sample. Only at the end of the session were the tasters informed about the purpose of the test and the differences between the samples.

### 2.5. Statistical Data Analysis

The data of NoV concentration in oysters are reported as median and interquartile range (IQR). We used the non-parametric test, Wilcoxon signed rank test, to evaluate the differences between the mean number of genomic copies determined before and after treatment with each hydrolate and control. All possible comparisons have been made. All parameters collected during the tasters’ evaluations were coded into binary (i.e., the type of tastes and flavours perceived) and ordinal categories (i.e., physical characteristics of the oysters, the degree of saltiness, sweetness, sourness and bitterness of the oysters, and the experts’ overall appreciation scoring). The Kruskal–Wallis rank test was used to assess differences between untreated oysters and oysters treated with hydrolates in the predefined ordinal variables. The Fisher exact test was applied to evaluate differences in the kinds of tastes and flavours reported by the experts’ panel regarding untreated and treated oysters.

Data were managed and analyzed with the use of the STATA 17 software, and the results were visualized with the use of the R free software (version 4.2.2). For all analyses, the statistical significance was set at *p* < 0.05.

## 3. Results

### 3.1. Chemical Composition of the Hydrolates

An SPME-GC-MS qualitative analysis identified the chemical composition of the hydrolates. The main components were carvacrol (58.67%), linalool (17.11%), and cymene (11.23%) in *T. serpyllum* hydrolate (Table 1); limonene (53.45%), beta-pinene (20.60%), and gamma-terpinene (14.03%) in *C. limon* hydrolate (Table 2).

### 3.2. Norovirus Quantification in Treated and Untreated Oyster Samples

**Figure 1:**
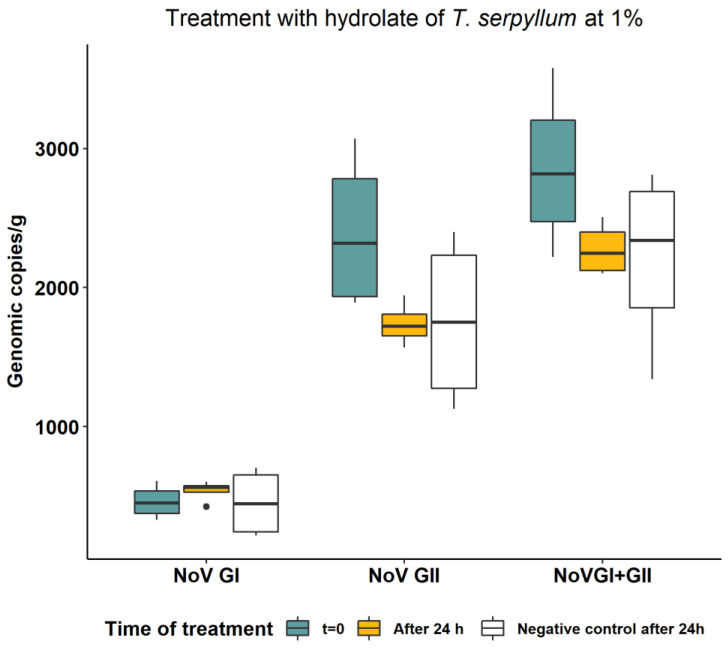
NoV RNA concentration in oysters before and after treatment of 24 h with hydrolate of *T. serpyllum* at 1%. Values are expressed in genomic copies/gram.

**Figure 2:**
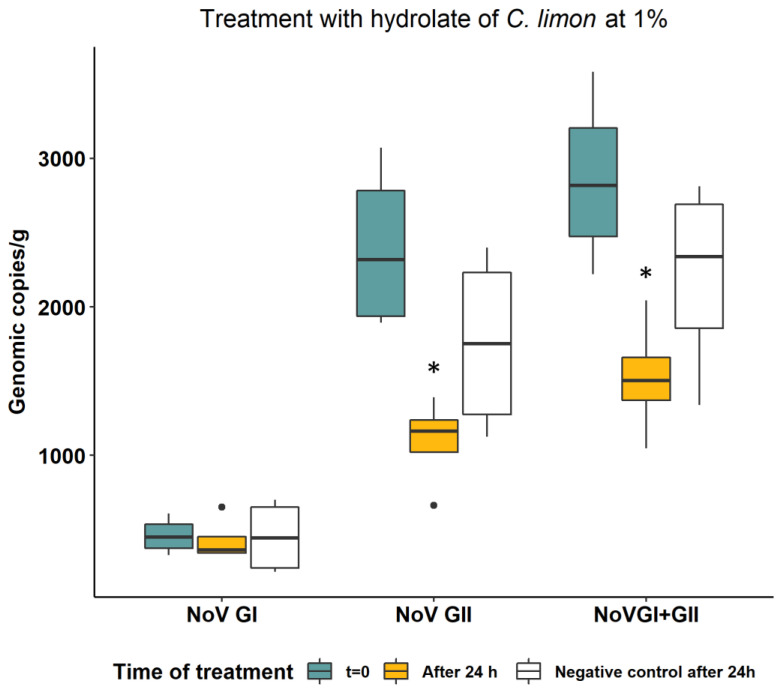
NoV RNA concentration in oysters before and after treatment of 24 h with hydrolate of *C. limon* at 1%. Values are expressed in genomic copies/gram. Boxplots indicated with asterisks were statistically significantly different with respect to t = 0 and negative controls (Wilcoxon signed rank test, *p* < 0.05).

We determined the virucidal efficacy of the hydrolates by comparing the reduction of RNA concentration of NoV in oysters before and after treatment with respect to controls (without hydrolates). The results for the hydrolates of thyme and lemon at 1% are shown in Figure 1 and Figure 2, respectively. After 24 h, the untreated negative control exhibited a natural decay of the NoV GII viral RNA by 26.8%, corresponding to a 0.14-log reduction. This reduction was not observed for NoV GI RNA (Figure 1 and Figure 2). In oysters treated with 1% of *C. limon* hydrosol for 24 h, the NoV concentration was significantly reduced (*p* = 0.0433), compared to the control, for both genogroup II (0.2 log reduction) and the sum of the two NoV genogroups (GI + GII) (0.15 log reduction), while no effect was observed on NoV GI concentration. Treatment with 1% thyme hydrosol for 24 h did not result in a reduction in NoV concentration compared with the untreated control for either genogroup.

### 3.3. Sensory Analysis

According to the tasters’ responses, the oysters treated with the hydrolates maintained similar sensory characteristics compared with the untreated oyster group. On average, the sensory profiling of treated and untreated oysters was generally described as having a marine flavour (n = 87; 82.9%) and a taste (n = 100; 95.2%) with hints of seaweed (n = 76; 72.4%) and at times a metallic note (n = 51; 48.6%). However, changes in the intensity of marine flavour were perceived in the treated oyster groups. In particular, the marine flavour seemed to decrease in intensity with the treatment, especially in oysters treated with *T. serpyllum* hydrolate (Fisher’s exact test, *p* < 0.05). Other characteristics, including citrus and thyme flavours and tastes, were also reported by the tasters, but these were unaffected by the treatment (Table 3; *p* > 0.05).

**Figure 3:**
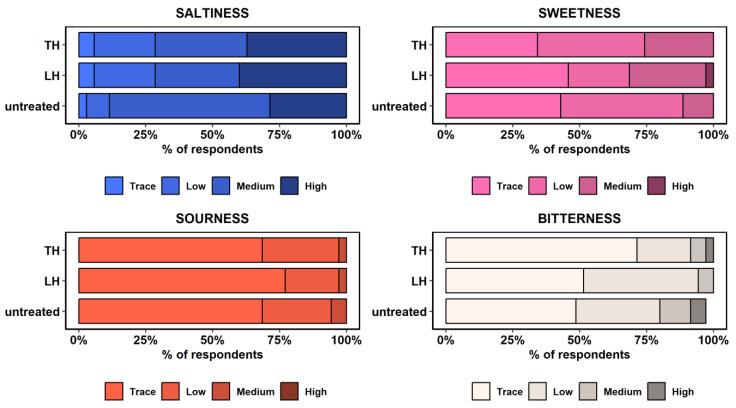
Perception of saltiness, sweetness, sourness, and bitterness levels in untreated oysters and oysters treated with *C. limon* hydrolate (LH) and with *T. serpyllum* hydrolate (TH). Note: The response level for the bitter taste category in untreated oysters is below 100% due to a missing response in one of the assessed oyster specimens.

Different levels of saltiness, sweetness, sourness, and bitterness were also reported by the tasters (Figure 3). Saltiness perception was generally reported as medium to high (n = 81; 77.1%), especially in the untreated group (88.6%). Similarly, oysters treated with *C. limon* hydrolate (71.4%) and with *T. serpyllum* hydrolate (71.4%) displayed comparable saltiness levels (Kruskal–Wallis, *p* > 0.05), while saltiness went unnoticed in 4.8% (n = 5) of the total oysters examined. By contrast, sweetness, sourness, and bitterness levels were comparable between treated and untreated oysters, being mainly described as low or imperceptible. Other taste patterns were reported, although not significantly; for instance, the levels of sweetness and bitterness tended to increase and decrease in treated oysters, respectively, while the sourness level remained unchanged regardless of the treatment (Figure 3; Kruskal–Wallis rank test, *p* > 0.05).

Furthermore, the treatments had no impact on the physical characteristics of the oysters. Both treated and untreated oysters generally displayed a greenish (n = 33; 31.4%) to greyish (n = 65; 61.9%) shade with varying levels of opacity, ranging from clear (n = 47; 44.8%) to opalescent (n = 41; 39.1%). A few tasters reported a whitish shade and cloudy opacity (n = 6; 5.7%), while 11 out of the 105 oysters assessed showed no changes in opacity. Additionally, the texture of oysters was described as tender (61.0%) or firm (31.4%), with only five specimens being characterized as mushy (Table 3). Nonetheless, no relation between the treatment and the observed differences in shade, opacity, and texture characteristics was detected (Kruskal–Wallis rank test, *p* > 0.05).

**Figure 4:**
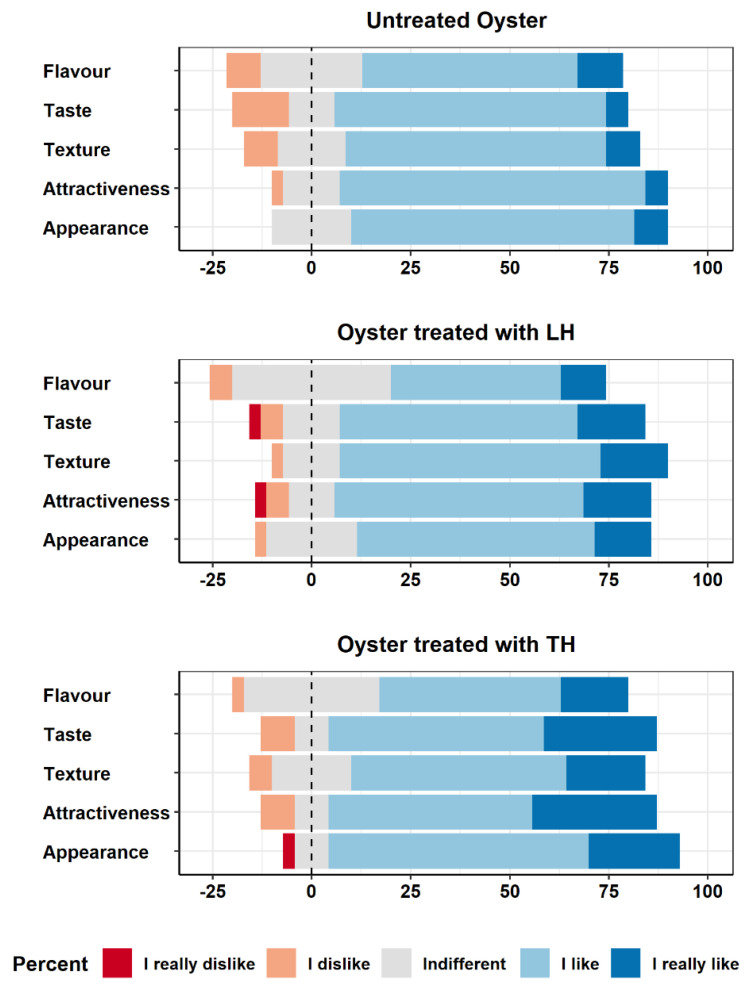
Overall sensory scores given by the tasters to untreated oysters and oysters treated with *C. limon* hydrolate (LH) and *T. serpyllum* hydrolate (TH).

In general, both treated and untreated oysters received positive evaluations from the tasters (Figure 4). With regard to the untreated oysters, some negative scores were mainly related to taste (considered unpleasant by 14.3% of the participants), texture, and smell (8.6% reported negative scores). Instead, a higher variability in scoring was found among the treated samples, where responses tended to be more neutral compared to those obtained for the untreated group. This result is particularly evident in flavour, where the percentage of neutral responses increased from 25.7% in the untreated group to 34.3% for oysters treated with *T. serpyllum* hydrolate and 40.0% for oysters treated with *C. limon* hydrolate. Moreover, the number of positive scores seemed to rise for both oysters treated with *C. limon* and *T. serpyllum* hydrolates, while the number of negative scores seemed to follow a downward trend. For example, this phenomenon was observed for taste (oysters treated with *C. limon* hydrolate: 77.1%, and *T. serpyllum* hydrolates: 82.9, vs. untreated: 74.3%) and texture characteristics (82.9% and 74.3% vs. 74.3%). However, these variations were statistically unsignificant (Kruskal–Wallis rank test, *p* < 0.05), indicating that the product characteristics in the treated and untreated groups can be considered comparable.

## 4. Discussion

For the first time, the hydrolates of *T. serpyllum* and *C. limon* were used to reduce the concentration of NoV during oyster depuration. The results revealed the effectiveness of *C. limon* hydrolate in reducing the concentration of NoV RNA, resulting in a 0.2 log reduction (corresponding to a mean reduction of 37.7%) compared to the negative sample, after 24 h of treatment. Interestingly, this reduction occurred only for NoV genogroup II while no effect in reducing the concentration of NoV RNA was observed for genogroup I. Therefore, the data obtained in this study demonstrate that the investigated hydrolates exhibit varying effects against the two genogroups of NoV. Previous studies have observed that different norovirus genotypes bind to different carbohydrate ligands present in oyster tissue. In particular, NoV genogroup I binds mainly to digestive tissues but not to other organs, while genogroup II binds to both digestive tissue and the gills, albeit to a lesser extent [40]. Depending on the ligand to which the two genogroups are bound, differences in the persistence of viral particles within the different organs may be observed. It has been hypothesized that the ligand recognized by GII can lead to a more rapid degradation or release, compared to the one recognized by GI, determining differences on the viral persistence within the body of the mollusks [40,41,42]. Based on this information, it could be hypothesized that the hydrolate of *C. limon* may affect noroviruses linked to gill tissues, which are in closer contact with the hydrolates during filtration, while it may not have the same effect on noroviruses inside the digestive tissue, possibly due to interactions with the oyster’s metabolism and digestive processes. In fact, genogroup I, which only binds to the digestive tissues, was not reduced by the treatment with the hydrosol.

In this study, the mode of action of hydrosols against NoV was not investigated. Previous studies have revealed that the virucidal mechanism of plant compounds on non-enveloped viruses can vary, including 1. degradation of the viral capsid or nucleic acid; 2. interference with the absorption of the virus on the host cells by binding to the surface of the virus; and 3. interference with the attachment of the viral surface protein to the cellular receptors [43,44,45,46].

Based on the chemical characterization performed, the *C. limon* hydrolate used in this study contains limonene and beta-pinene as primary components. These compounds have demonstrated antiviral action in previous studies against both non-enveloped viruses such as norovirus [47] and enveloped viruses [34]. Also, the thyme hydrolate used in this study contains a high relative concentration of carvacrol (58.67%), a compound with known antiviral activity [35,36,37]. However, in this study the thyme hydrolate was not effective in reducing NoV concentration. In our previous in vitro study, where we evaluated the virucidal efficacy of *T. serpyllum* and *C. limon* hydrolates against MNV, a surrogate of NoV, we observed a reduction in viral infectivity of approximately 99% (2.9 log TCDI_50_/mL) for thyme and 92% (1.8 log TCDI_50_/mL) for lemon. Furthermore, thyme showed faster virucidal activity than *C. limon*. The infectivity of MNV immediately decreased by 2 log after treatment with 1% *T. serpyllum* hydrolate, while the reduction in MNV after treatment with 1% *C. limon* hydrolate was about 1 log immediately and an additional 1 log after 24 h treatment [33]. Despite using the same hydrolates at the same concentration, the results obtained in the in vivo application for the reduction in NoV in oysters were notably different. However, these differences are not surprising for several reasons. First, the virus under investigation is different, even though MNV is a surrogate for human NoV. Other authors stated that the antiviral activity of natural compounds is closely linked to the type of virus investigated [48,49]. Second, the antiviral activity of essential oils and plant extracts is normally lower in food matrices, compared to in vitro tests [50]. Virions present in food matrices and foodstuffs were found to be more resistant to the antiviral activity of plant compounds, compared with virions present in water [51,52]. Moreover, in our study, the treated food was alive, introducing additional factors, such as the physiology and metabolism of the mollusk, that can influence the effectiveness of the substance studied, but also the medium (sea water) into which the hydrolates were added. The antiviral studies were performed in tissue culture mediums or sterile buffer solutions, which may not fully simulate the practical scenarios.

A potential limit in the practical application of hydrolates in foods could be their taste and odor, as they have the potential to alter the sensory properties of foods. To address this concern, our study included a sensory analysis conducted with a panel of 36 tasters to assess the impact of hydrolate treatments on the oysters’ taste and odor. The sensory analysis data demonstrated that the hydrolate treatment did not affect the taste and/or odor of the oysters. Unexpectedly, the only significant modification caused by the treatment with the hydrolates was a decrease in the intensity of the marine flavour, especially in the oysters treated with the thyme hydrosol. However, this change actually improved the overall taste and acceptability of the oysters. In fact, many tasters did not prefer the very salty taste of the untreated Pacific oysters, as they are raised in sea waters with high salinity, unlike other oysters present on the market.

## 5. Conclusions

The data obtained in this study suggest that the addition of hydrosols during the depuration process can be a promising strategy to enhance both the safety and sensory characteristics of Pacific oysters. Using hydrolates in shellfish depuration offers advantages over other treatments. These hydrolates are natural phytocomplexes that are cost-effective and highly biodegradable, and carry a low risk of toxicity or side effects [32,55]. Therefore, they are excellent candidates for use as processing aids in oyster depuration. This in vivo study, conducted under standard depuration conditions, demonstrates that *C. limon* and *T. serpyllum* hydrolates do not alter the sensory characteristics of the oysters, but may even improve their taste and overall acceptability.

These results can be a starting point for further research on the virucidal activity of hydrosols on NoV present in food, even if the reduction obtained in our study was quite low. To improve the effectiveness of the treatment, it may be worthwhile to explore combinations of hydrolates with other treatments. Further studies should focus on improving purification performance, elucidating the mechanisms of action against NoV, and determining whether different hydrosol concentrations may yield different reductions in NoV. Moreover, although the active compounds in the hydrolates are highly diluted, any possible toxicological effect should be investigated, to confirm that these substances can be safely used as processing aids in food production. In conclusion, this study opens up several perspectives for the development of prophylactic tools for oyster purification and for mitigating the risks associated with NoV exposure in food.

## Figures and Tables

**Table 1:** Chemical composition of *Thymus serpyllum* hydrolate. RI cal: calculated retention index; RI ref: retention index from the literature (source: Pherobase).

No. Peak	Compound	RI Cal.	RI Ref.	Content (%) ^1^
1	Alpha-pinene	937	939	1.25
2	Camphene	952	953	0.31
3	Beta-pinene	979	975	0.26
4	Beta-myrcene	992	991	2.02
5	Alpha-phellandrene	1005	1005	0.39
6	Cymene	1028	1026	11.23
7	Gamma-terpinene	1063	1062	6.04
8	Linalool	1103	1101	17.11
9	Carvacrol	1315	1317	58.67
10	Caryophyllene oxide	1593	1583	2.73

^1^ %: results are expressed as a percentage of the total chromatographic area.

**Table 2:** Chemical composition of *Citrus limon* hydrolate. RI cal: calculated retention index; RI ref: retention index from the literature (source: Pherobase).

No. Peak	Compound	RI Cal.	RI Ref.	Content (%) ^1^
1	Alpha-pinene	937	939	3.12
2	Beta-pinene	981	975	20.60
3	Beta-myrcene	992	991	2.72
4	Limonene	1039	1031	53.45
5	Gamma-terpinene	1065	1062	14.03
6	Terpinolene	1090	1089	0.70
7	Linalool	1100	1101	0.30
8	Terpinen-4-ol	1181	1178	0.10
9	Alpha-terpineol	1193	1189	0.35
10	Neral	1245	1242	1.28
11	Geranial	1274	1271	2.24
12	Beta-bisabolene	1512	1509	1.02

^1^ %: results are expressed as a percentage of the total chromatographic area.

**Table 3:** Number (and percentages) of participants who perceived the reported sensory characteristics in the untreated oysters and oysters treated with *C. limon* hydrolate (LH) and with *T. serpyllum* hydrolate (TH).

		Untreated Oyster	Oyster LH	Oyster TH
*Physical characteristics*				
Shade				
	Whitish	3 (8.6)	1 (2.9)	2 (5.7)
	Greenish	12 (34.3)	12 (34.3)	9(25.7)
	Greyish	20 (57.1)	21 (60.0)	24 (68.6)
Opacity				
	Clear	16 (45.7)	16 (45.7)	15 (42.9)
	Opalescent	16 (45.7)	12 (34.3)	13 (37.1)
	Cloudy	-	3 (8.6)	3 (8.6)
Texture	Mushy	1 (2.9)	2 (5.7)	2 (5.7)
	Tender	26 (74.3)	20 (57.1)	18 (51.4)
	Firm	8 (22.9)	11 (31.4)	14 (40.0)
*Sensory characteristics*				
Flavour (yes/no)				
	Marine	33 (94.3) *	28 (80.0)	26 (80.0) *
	Citrus	4 (11.4)	3 (8.6)	3 (8.6)
	Garlic	5 (14.3)	5 (14.3)	3 (8.6)
	Rosemary	2 (5.71)	5 (14.3)	3 (8.6)
	Thyme	3 (8.6)	6 (17.1)	5 (14.3)
Taste (yes/no)				
	Marine	34 (97.1)	33 (94.3)	33 (94.3)
	Seaweed	28 (80.0)	23 (65.7)	25 (71.4)
	Fruity	1 (2.9)	0	2 (5.71)
	Metallic	19 (54.3)	19 (54.3)	13 (37.1)
	Thyme	4 (11.4)	4 (11.4)	7 (20.0)
	Spinach	7 (20.0)	8 (22.9)	7 (20.0)
	Citrus	5 (14.3)	7 (20.0)	2 (5.71)

* Percentages indicated in the same row with an asterisk are statistically different (Fisher’s exact test, *p* < 0.05).

## Data Availability

The data used to support the findings of this study can be made available by the corresponding author upon request.
